# How Seeds Attract and Protect: Seed Coat Development of *Magnolia*

**DOI:** 10.3390/plants13050688

**Published:** 2024-02-29

**Authors:** Qiuhong Feng, Ming Cai, Honglin Li, Xin Zhang

**Affiliations:** 1 Ecological Restoration and Conservation on Forest and Wetland Key Laboratory of Sichuan Province, Sichuan Academy of Forestry, Chengdu 610081, China; fqiuhong@163.com; 2 Beijing Key Laboratory of Ornamental Plants Germplasm Innovation & Molecular Breeding, National Engineering Research Center for Floriculture, Beijing Laboratory of Urban and Rural Ecological Environment, Beijing Forestry University, Beijing 100083, China; jasoncai82@163.com; 3 College of Forestry, Southwest Forestry University, Kunming 650224, China; lihonglin06@163.com; 4 College of Forestry, Northwest A&F University, Yangling 712100, China

**Keywords:** ovule, magnoliaceae, sarcotesta, sclerotesta, morphology, development

## Abstract

Seeds are one of the most important characteristics of plant evolution. Within a seed, the embryo, which will grow into a plant, can survive harsh environments. When the seeds are mature, the mother plant will disperse them from its body, allowing them to be taken away to grow in a new place. Otherwise, if the young generation grows alongside the mother plants in the same place, they will compete for sunlight and nutrition. The mother plants use different strategies to send away their seeds. One of these strategies is endozoochory, which means that the seeds disperse via ingestion by animals. There is a conflict between the seeds’ abilities to attract animals and protect the embryo within the digestion systems of animals. *Magnolia* seeds exhibit typical endozoochory. The seed coats of *Magnolia* feature sarcotestas and sclerotestas. The sarcotesta, which is fleshy, bright-colored, and edible, attracts animals. The sclerotesta is hard and woody, protecting the embryo from the digestive systems of animals. In this study, we used scanning electron and light microscopes to examine the development of the sarcotesta and sclerotesta of *Magnolia stellata* seed coats. The results showed that the sarcotesta and sclerotesta come from the outer integument. This result confirms the hypothesis of Asa Gray from 1848. The dependence of the seed dispersal strategy on structural development is discussed.

## 1. Introduction

Seeds are one of the most important characteristics in plant evolution. Within a seed, an embryo, which will grow into a plant, can survive harsh environments. When seeds are mature, the mother plant will disperse them from its body, allowing them to be taken away to grow in a new place by themselves or with others. Otherwise, if the young generation grows alongside the mother plant, they will compete for sunlight and nutrition. The mother plants use different strategies to disperse their seeds. One of these strategies is endozoochory, whereby seeds disperse via ingestion by animals. The seeds of *Magnolia* represent a special case that has been studied throughout the history of endozoochory.

Gray [1] first described how the seeds of *Magnolia* are not arillate, but baccate, or, in other words, that the fleshy coat is the testa. In 1856, there was a debate between Miers and Gray, the two most famous botanists who were contemporaries of Darwin, about whether the fleshy structure of these seeds was an aril or a testa [2]. Miers criticized Gray, stating that “the external fleshy coat of the seeds of *Magnolia* is described as testa and its thick bony shell as tegmen, or inner integument, the true integument having escaped the notice of that excellent botanist”, and “there is no reason to doubt that, in *Magnolia*, the scarlet envelope is due to a subsequent growth over the primine”; therefore, the fleshy coat is an arillus [2]. Gray [3] settled three points by direct observation. First, no accessory cover or arillus develops over or upon the primine of the ovule. Second, the fleshy envelope of the seed represents the primine or outer coat of the ovule. Third, the bony coat of the seed is represented in the ovule only by the innermost layer of young cells, lining the primine. During seed growth, the meristematic cells divide, and their walls irregularly elongate, thicken, and harden, forming a crustaceous or bony coat, so the seed character is best expressed as “drupaceous”. These results were substantiated by Maneval [4] and Earle [5]. Kapil and Bhandari [6] found the same structure as Earle [5] in different species; however, there are two integuments in *Magnolia* ovules. If the outer integument develops into a sarcotesta and sclerotesta, where is the inner integument? Boer and Bouman [7] observed the integument development of *Magnolia stellata* and *Magnolia virginiana*, discovering that the inner integument only became thicker by “secondary” cell division in the micropylar region, and this does not contribute substantially to the formation of the testa, which persists as a “papery” layer. However, Corner [8] pointed out that the lignified cells of the endotesta are peculiar, and electron microscopy is required for elucidation, which provides a higher resolution than an ordinary microscope. Matsui et al. [9] investigated the ovular development and morphology of some Magnoliaceae species with SEM and a microtome. They found that the outer integument and the funicular outgrowth form an envelope complex. Yamada et al. [10] rechecked the outer integuments and funicular outgrowth complexes in the ovules of *Magnolia grandiflora*. The interpretation of a single cupular outer integument was not supported.

Here, Corner’s advice was followed to check the late-lignified cells of the endotesta and the inner integument around the micropylar region of mature seeds. In this study, we used a microtome, scanning electron and light microscopes to examine the development of the sarcotesta and sclerotesta of *Magnolia stellata* seed coats. We aimed to confirm the origin of sarcotestas and sclerotestas in *Magnolia* and to discuss the dependence of the seed dispersal strategy on structural development.

## 2. Material and Methods

### 2.1. Material Collection and Preparation Magnolia stellata

Plant material from *Magnolia stellata* was sampled from a shrub about 3–4 m high in the Botanical Garden of the Ruhr University Bochum.

**Figure 1:**
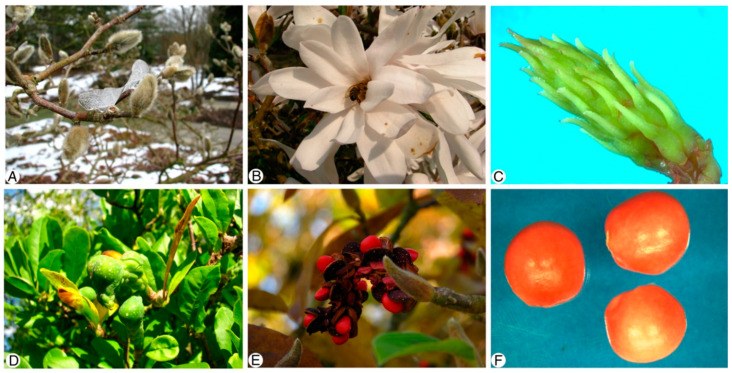
The flower buds, flower, androecium, young fruit, mature fruit and seeds of *Magnolia stellata.* (**A**)—flower buds; (**B**)—flowers; (**C**)—follicetum, (**D**)—young follicles; (**E**,**F**)—seeds.

The flower buds (Figure 1A), flowers (Figure 1B), follicetum (Figure 1C), young follicles (Figure 1D), and seeds (Figure 1E,F) were continuously taken from October 2009 to May 2012 once a week during the growing season. The ovule begins to develop at the end of the summer, and the entire flower is hidden in an approximately 5 mm long terminal flower bud (Figure 1A). The flower opens the following spring (Figure 1B).

Samples at different developmental stages were collected from living plants and dissected in 70% ethyl alcohol. The selected structures were analyzed under a dissection microscope (ZEISS Stemi SV 11, Jena, Germany) and photographed with a KEYENCE VHX-500F digital microscope (Neu-Isenburg, Germany). After this, the material was fixed in FAA (formalin/acetic acid/ethyl alcohol 70% = 5:5:90) under a moderate vacuum for at least half hour. After two days, the FAA was replaced with 70% ethyl alcohol for storage.

### 2.2. Scanning Electron Microscopy (SEM)

The structures of interest were transferred from ethyl alcohol to dimethoxymethane and stored at 4 °C for at least 48 h. Dimethoxymethane chemically dehydrates plant tissues and serves as an intermedium in the drying process. Drying was performed using a CPD 030 (BALZERS, Bingen am Rhein, Germany). Depending on the size and structure of the material, the dried tissue was mounted on aluminum stubs either with conductive pads (Leit Tabs, PLANO, Wetzlar, Germany) or conductive carbon cements (Leit-C, PLANO, Germany), and then stored in a desiccator with silica gel.

The samples were sputter-coated with gold for 200–400 s at 42–43 mA (BAL-TEC SCD 050, Amstetten, Germany). Scanning electron microscopy was performed with a DSM 950 (ZEISS, Germany). For documentation, a digital image processing system (DIPS 2.2, POINT ELECTRONIC, Halle, Germany) was used, allowing for storage in the tiff format (2000 × 2000 pixels). The size was adapted to the plate format using Adobe Photoshop^®^ (additional image processing was not performed).

### 2.3. Anatomical Studies

The anatomical studies were performed using classical paraffin serial sectioning. The fixed material was transferred from FAA to 70% ethyl alcohol for at least 24 h, and then gradually transferred to alcohol and Paraplast Plus^®^ (McCORMICK SCIENTIFIC, Hunt Valley, MD, USA) according to Gerlach ([11], modified).

The embedded samples were mounted on wooden stubs and cut with a rotary microtome (LEICA RM 2065). Serial sections (5–12 µm) were mounted on microscope slides with protein glycerol and stored at 40 °C for at least 12 h. After staining with Safranin/Astra Blue, according to Gerlach (1984, modified), the slides were covered with Entellan^®^NEW (MERCK, Darmstadt, Germany) and glass. Photographs were taken with a ZEISS Axioplan using a Color View II camera (OLYMPUS, Tokyo, Japan). For multiple image alignment, the software Cell^F (OLYMPUS 5.0) was used. To produce schematic drawings, the software CorelDRAW X4 2.0 was used.

## 3. Results

### Ovules and Seed Coats of Magnolia stellata

**Figure 2:**
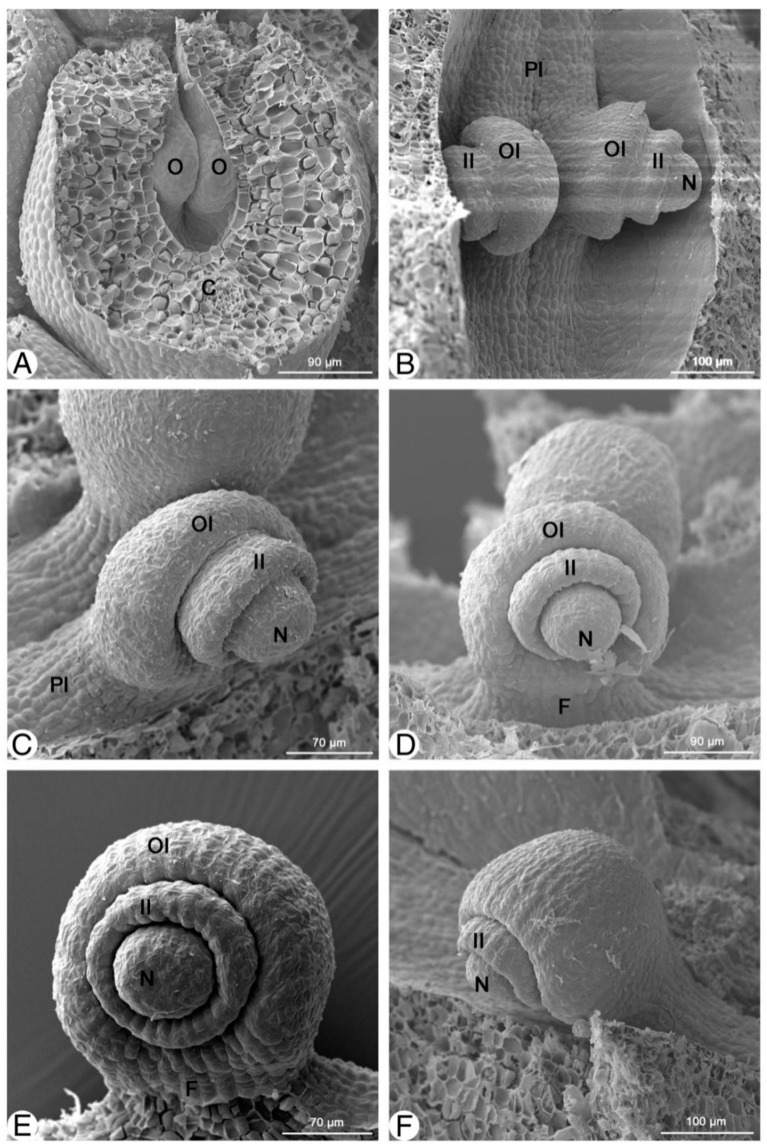
Ovular development of *Magnolia stellata.* (**A**)—two ovule primordia per locule; (**B**)—young ovule with inner and outer integuments developing; (**C**–**F**)—the development of inner and outer integuments before nucellus covering and ovule incurvation. C—carpel; F—funiculus; II—inner integument; N—nucellus; O—ovule; OI—outer integument; Pl—placenta.

In early September, each carpel has two ovule primordia on the marginal placenta in the basal portion of the carpel, which is adnate to the floral receptacle (Figure 2A). The ovule primordium is conical, dorsoventrally compressed, and undifferentiated along the placenta. Until the end of September, the carpels and ovule primordia change neither in size nor developmental stage. Nevertheless, some of the only slightly more developed carpels on the basal portion of the floral axis contain ovule primordia with incipient differentiation.

Comparing the well-developed ovules sampled in mid-December to those taken at the beginning of February in the following year, it was found that *Magnolia stellata* rests during winter. At the beginning of March, the ovule’s inner and outer integuments visibly differentiate (Figure 2B). In the SEM photo of a two-week-old ovule, the inner integument forms a ring around the nucellus, and the outer integument is more or less around the inner integument and nucellus (Figure 2C,D). The nucellus protrudes in the middle. Because of the development of the ring-shaped outer integument, the width of the ovules doubles over a period from four to six weeks (Figure 2E,F).

**Figure 3:**
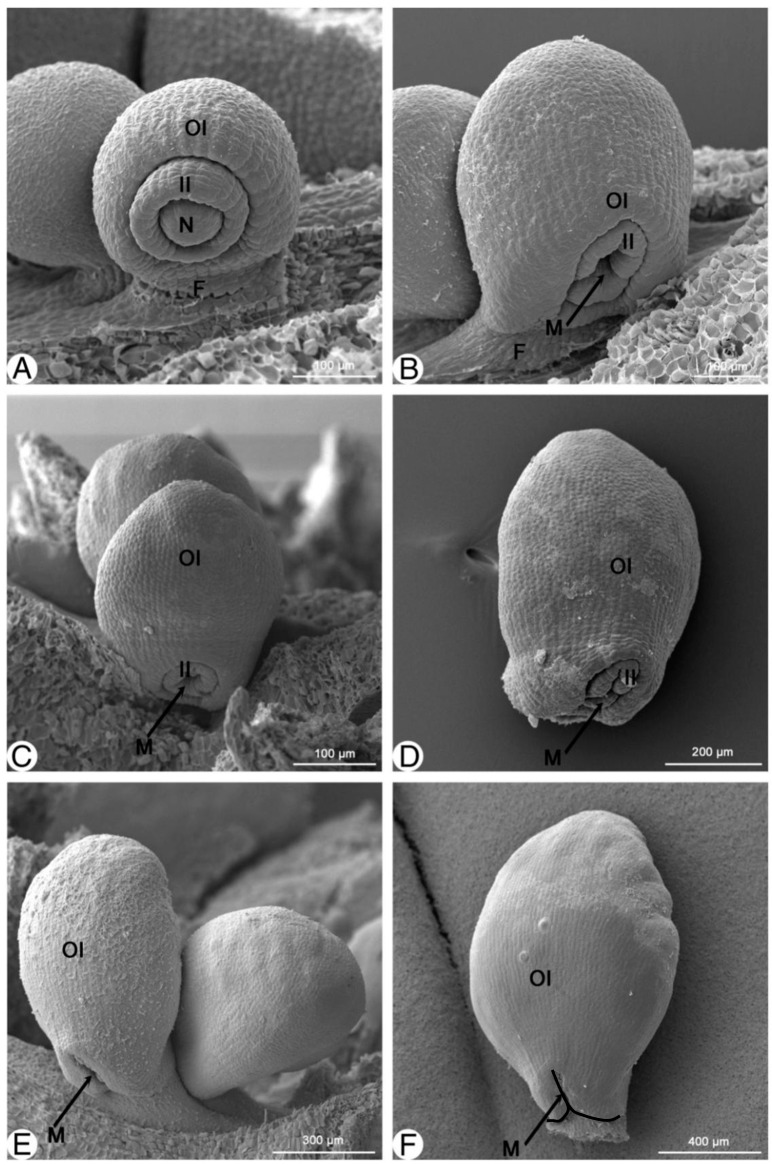
Stages of micropyle development of *Magnolia stellata.* (**A**)—the outer integument half-covering the inner integument; (**B**)—the outer integument covering the nucellus and inner integument with some lobes on the tip; (**C**)—the hood-shaped outer integument; (**D**–**F**)—the 人-shaped micropyle forming. F—funiculus; II—inner integument; M—micropyle; N—nucellus; OI—outer integument.

In mid-March, the inner integument is as enlarged as the annular rim; the outer integument is semi-annular and slightly interrupted on the concave side by the funiculus (Figure 3A). Later, peri- and anticlinal divisions occur on the concave side, and an outgrowth forms.

As the ovule is further incurved, the elongated outer integument surrounds the inner integument to an increasing extent. The inner integument becomes irregularly lobed at the distal edge (Figure 3B).

**Figure 4:**
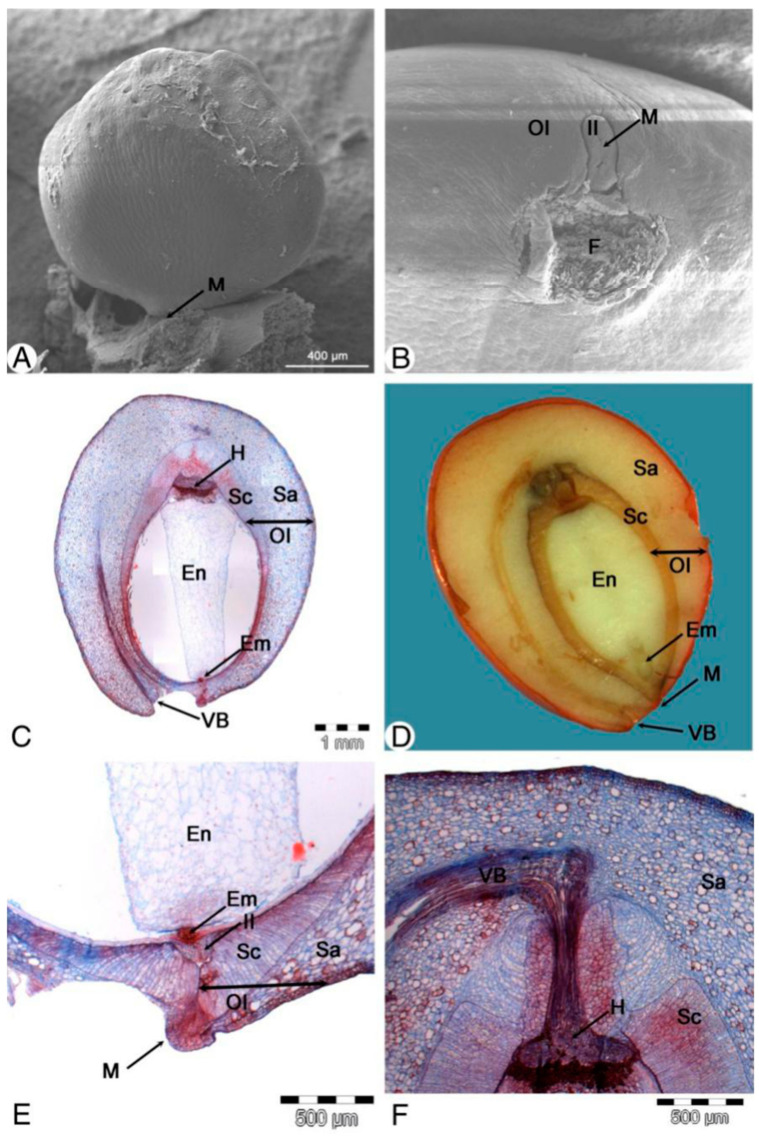
Seed morphology and anatomy of *Magnolia stellata.* (**A**)—a mature ovule; (**B**)—a micropyle of (**A**); (**C**)—the longitudinal section of the seed with an embryo; (**D**)—the hand cutting of a seed after stage (**C**); (**E**)—magnification of the micropyle portion of (**C**) to show the inner integument tissue left on the micropyle and outer integument that has developed into a sarcotesta and sclerotesta; (**F**)—the chalaza portion of (**C**) to show the vascular bundle and hypostase. Em—embryo; En—endosperm; H—hypostase; F—funiculus; II—inner integument; M—micropyle; OI—outer integument; Sa—sarcotesta; Sc—sclerotesta; VB—vascular bundle.

When the ovule is more incurved at a later stage, the outer integument overgrows the inner integument (Figure 3C). The lateral micropylar lobes of the outer integument are decurrent to the lateral sides of the funiculus, and it becomes obvious that the outer integument is hood-shaped (Figure 3D). The opening of the outer integument changes from a round shape to a collar shape at the top. This is because of the elongation of the lateral portion rather than the middle part (Figure 3E). A micropyle is formed by the two neighboring distal lobes of the outer integument, and the ovule is then further elongated and becomes anatropous. At maturity, the exostome develops an inverted 人 shape, which is a transverse slit with a middle notch that emerges due to the collar shape opening at an earlier stage (Figure 3F and Figure 4A,B).

At the end of September, in the mature seeds, the outer integument is differentiated into two layers, namely an outer fleshy one, which is filled with oil vacuoles, and an inner stony layer (Figure 4C,D). The inner integument only forms a thin layer in the ripe seeds (Figure 4E,F).

## 4. Discussion

Seeds develop from a fertilized ovule, and seed coats develop from integuments that protect the embryo sac, which contains the egg [8,12]. Only one hole, called a micropyle, is left for the pollen tube to enter the embryo sac to complete fertilization. After fertilization, the ovule transforms and differentiates into a seed, and the integuments become a seed coat, which is a stable and closed container, to allow the embryo to survive rather long phases of dormancy [12]. Integument and seed coat development results in two sets of problems, each giving rise to new evolutionary constraints. First, after fertilization, the micropyle should be closed. The simplest way to achieve this is by shrinking; however, this only works under dry conditions. This result may explain why fleshy seed appendages originate from the micropyle or the funiculus. The more sophisticated the opening mechanisms are in regard to achieving germination, the more perfectly the seed coat encloses the embryo. However, after the seed matures, how is the seed transported far from the mother plant? There are many strategies for seed plant dispersal [13]; one of them is called endozoochory, which means the seed is dispersed by an animal. The seeds of *Magnolia* undergo endozoochory. To achieve endozoochory seed dispersal, the seeds need fleshy tissue to attract a hungry animal, a process that benefits the animals. Meanwhile, seeds must also protect the embryo by defending against digestive fluids in the stomach.

The observations of seed development showed that the sarcotesta and sclerotesta of *Magnolia stellata* come from the outer integument, which confirms the hypothesis of Asa Gray from 1848. The second (outer) integument always develops later than the first (inner) integument in angiosperms, and it is problematic to regard the second integument as a protective structure. If more protection is needed, the layer becomes thicker. Therefore, the evolutionary relevance of the outer integument is most likely different from that of the inner integument. An underdeveloped second integument is not protective, and the stepwise evolution of this feature seems problematic if only the final structure is functional. It is more likely that the outer integument is equivalent to the aril of Taxaceae and Podocarpaceae in Gymnosperms, and this aril also develops later than the first (inner) integument [14].

The micropyle may be open, forming an open canal, or closed. In the former, the micropyle may be sealed by a secretion [15,16,17]. In the latter, a closed pollen-transmitting tract is formed by post-genital fusion [18]. Thus, this diversity is analogous to the carpels sealed by secretion or post-genital fusion [19]. However, details of the histology of mature micropyles have only rarely been studied [20].

The aril is one of the best-known and most widespread fleshy seed appendages [21] for endozoochory. An aril is any specialized outgrowth from the funiculus that covers or is attached to the seed. Arils occur in gymnosperms as well as in angiosperms. A typical gymnosperm aril starts to develop markedly later than the integument. The development of integuments and arils in gymnosperms is the same as those of the inner and outer integuments in angiosperms. Usually, both integuments in angiosperms are regarded as protective structures. However, it seems much more feasible during evolution to make one integument stronger than to add a second one, starting with its development from the ovular base. Therefore, it makes sense to assume that the outer integument in angiosperms did not originally protect seeds. Angiosperms, which are in need of a fleshy seed coat, would have to regain such a structure, according to Dollo’s principle, in a different way or through a separate structure.

## 5. Conclusions

*Magnolia* seeds exhibit typical endozoochory. The seed coats have sarcotestas and sclerotestas. The sarcotesta, which is fleshy, bright-colored, and edible, attracts animals. The sclerotesta is hard and woody, protecting the embryo from the digestive systems of animals. Both the sarcotesta and sclerotesta originate from the outer integument, thus confirming the hypothesis of Asa Gray from 1848.

## Data Availability

The data presented in this study are available on request from the corresponding author.
